# Descompensação de Insuficiência Cardíaca por Arbovirose

**DOI:** 10.36660/abc.20180316

**Published:** 2020-05-11

**Authors:** Carolina Cunto de Athayde, Fabio Akio Nishijuka, Márcia Cavalcanti de Campos Queiroz, Monica Medeiros Luna, Jaime Lobo Figueiredo, Nadia Matias de Albuquerque, Sebastião Carlos Ribeiro de Castilho, Renata R. T. Castro

**Affiliations:** 1 Hospital Naval Marcilio Dias Rio de JaneiroRJ Brasil Hospital Naval Marcilio Dias – Cardiologia, Rio de Janeiro , RJ - Brasil; 2 Brigham and Womens Hospital Boston EUA Brigham and Womens Hospital – Medicine, Boston – EUA

**Keywords:** Insuficiência Cardíaca/fisiopatologia, Recusa do Paciente ao Tratamento, Vacina Pneumocócica, Infecções por Arbovirus, Infecção por vírus Zika, Febre de Chikungunya

## Introdução

A insuficiência cardíaca (IC) é uma condição crônica com prevalência elevada e crescente no mundo. ^1 - 3^

A identificação da causa de descompensação da IC é extremamente importante para a correta condução dos casos, uma vez que poderá permitir o tratamento específico e prevenir novas internações. No Brasil, as principais causas de descompensação de IC são má aderência ao tratamento medicamentoso (30%) e infecções (23%), ^1^ principalmente as bacterianas pulmonares. ^4^ Por isso, pacientes com IC devem receber a vacina anti-pneumococo. Apesar de menos frequente, a descompensação pode dar-se por infecções virais, fato que justifica a vacinação contra influenza nestes pacientes. ^3^

Nos últimos anos diversas cidades brasileiras foram acometidas por epidemias de arboviroses antes consideradas raras como as causadas pelo vírus da Zika e da febre chikungunya. ^5^ Tais epidemias chamaram atenção da comunidade científica não só pelo número de pacientes atingidos, mas principalmente pelas frequentes sequelas, como microcefalia em filhos de gestantes acometidas pelo vírus da Zika e pelas artralgias incapacitantes e crônicas secundárias à febre de chikungunya. Apesar de existirem relatos de miocardite causada por arboviroses, ^6 - 8^ pouco se sabe a respeito dos riscos e complicações quando estas acometem pacientes que já têm diagnóstico de IC. A elevada prevalência de IC e a alta incidência de arboviroses no Brasil justificam o relato de caso a seguir.

## Relato do Caso

Paciente masculino de 71 anos, aposentado, procurou serviço de emergência com queixa de dispneia aos pequenos esforços com piora progressiva nos últimos dois dias evoluindo para dispneia em repouso e dispneia paroxística noturna após episódio de febre não aferida na véspera. Negou tosse, dor torácica, tonteira e síncope. O paciente tinha diagnósticos de miocardiopatia dilatada de etiologia hipertensiva/alcoólica, insuficiência renal crônica não-dialítica, fibrilação atrial permanente, hiperuricemia, doença pulmonar obstrutiva crônica e colelitíase. Era etilista e ex-tabagista (47 maços/ano, estando abstêmio há 6 anos). Fazia uso regular de carvedilol (12,5 mg pela manhã e 25 mg à noite), hidralazina (25 mg, 3 vezes ao dia), anlodipino (5 mg ao dia), furosemida (40 mg, 4 vezes ao dia), digoxina (0,125 mg ao dia), apixabana (2,5 mg, 2 vezes ao dia), bamifilina (300 mg ao dia) e formoterol/budesonida (12 mcg + 400 mcg, 2 vezes ao dia). Ao exame admissional, o paciente apresentava pressão arterial de 110 x 84 mmHg, frequência cardíaca de 86 bpm, frequência respiratória de 26 irpm e turgência jugular patológica a 30°. A ausculta pulmonar revelava murmúrios vesiculares universalmente audíveis, sem ruídos adventícios, e à ausculta cardíaca havia ritmo irregular, com bulhas normofonéticas, sem bulhas acessórias. Havia edema de membros inferiores (2+/4+) e não havia ascite ao exame físico.

Não havia evidência clínica de angina, novas arritmias ou infecção ( Tabela 1 ). O paciente e sua esposa negavam má-adesão ao tratamento medicamentoso, libação alcoólica ou ingestão excessiva de sal ou líquidos. Portanto, não foi possível identificar fatores precipitantes para o quadro de IC descompensada. O eletrocardiograma de admissão do paciente mostrou ritmo de fibrilação atrial e bloqueio de ramo esquerdo. Não havia alteração eletrocardiográfica sugestiva de isquemia miocárdica. A radiografia de tórax mostrou aumento do índice cardiotorácico, com discreta congestão pulmonar e ausência de derrame pleural ou consolidação pulmonar. Visando rastreamento infeccioso foi realizado exame de sedimento urinário, com resultado normal.

**Tabela 1:** – Resultados de exames laboratoriais durante a internação

Exames laboratoriais	Dias de internação				

	1	3	5	12	14
Proteína C reativa (mg/dL)	3.00	2.90	2.56	10.60	10.30
Ureia (mg/dL)	96	148	102	72	107
Potássio (mEq/L)	6.3	5.8	3.5	4.6	4.6
Creatinina (mg/dL)	2.0	3.6	2.2	1.6	1.9
Hemoglobina (g%)	14.9	16.3	14.0	14.1	13.0
Leucócitos (mil/mm ^3^ )	6.9	7.9	5.7	8.7	5.7
Plaquetas (mil/mm ^3^ )	106	135	87	80	111

**Figura 1:**
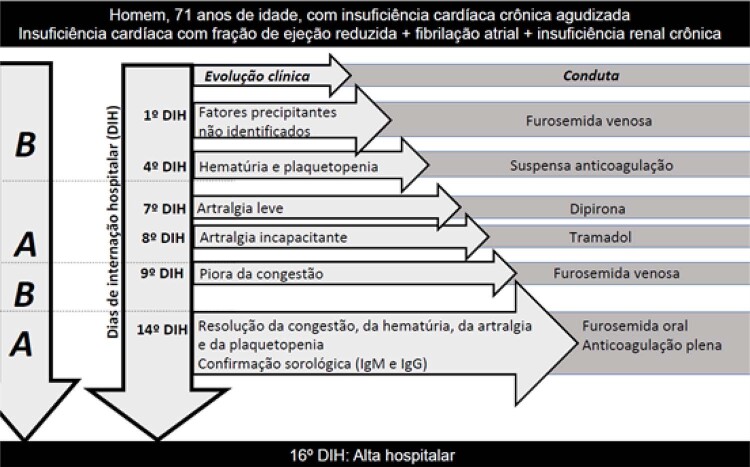
- Linha do tempo do relato de caso.

O paciente foi admitido, classificado com perfil hemodinâmico B ^9^ e submetido ao tratamento com diuréticos intravenosos ( Figura 1 ).

No terceiro dia de internação, o paciente evoluiu com piora da função renal, com clearance de creatinina (Cockroft-Gault) de 19 ml/min (creatinina 3,0 mg/dL) e hipercalemia (6,1 mEq/l). Devido a tal complicação, foi suspensa a digoxina. Após cinco dias, o paciente alcançou perfil hemodinâmico A, e optou-se por alterar a via de administração da furosemida de venosa para oral. Neste mesmo dia o paciente apresentou hematúria macroscópica, sendo suspensa a anticoagulação. No sétimo dia de internação o paciente queixou-se de artralgia leve, em joelhos e tornozelos, que associou à sua posição no leito. A despeito do uso de dipirona, no dia seguinte houve piora dos sintomas, com artralgia bilateral, de forte intensidade em joelhos, tornozelos, punhos e cotovelos, restringindo a movimentação no leito. O paciente não apresentou, dispneia ou precordialgia e mantinha perfil hemodinâmico A. Houve discreto controle álgico com uso regular de tramadol e no dia seguinte os exames laboratoriais revelaram plaquetopenia (queda de 135.000 para 87.000 por mm ^3^ em 5 dias - Tabela 1 ). Artralgia e plaquetopenia levantaram a suspeita de arbovirose e por isso foram solicitadas sorologias para dengue e chikungunya. O paciente evoluiu com piora da congestão periférica e central, com edema de membros inferiores (3+/4+), turgência jugular patológica, refluxo hepatojugular, estertoração crepitante bibasal e onda quadrada da pressão arterial sistólica à manobra de Valsalva. Não havia sinal de hipoperfusão e o paciente foi novamente classificado com perfil hemodinâmico B, sendo re-iniciada diureticoterapia venosa.

No 14° dia de internação já havia melhora da plaquetopenia, da artralgia e do padrão de congestão (com retorno ao perfil hemodinâmico A). Os exames laboratoriais não revelavam distúrbios eletrolíticos ( Tabela 1 ), ou alterações da função hepática. As sorologias para febre chikungunya foram positivas (IgG e IgM). Assim, foi reiniciada anticoagulação plena com apixabana, trocada analgesia regular por apenas se necessária. Durante toda a internação o padrão eletrocardiográfico foi mantido. No dia seguinte permaneceu estável, mantendo perfil hemodinâmico quente e seco, recebendo alta hospitalar com a seguinte prescrição: furosemida (40 mg, 2 vezes ao dia, carvedilol (25 mg, 2 vezes ao dia), atorvastatina (20 mg ao dia), formoterol + budesonida (12/400 mcg, 2 vezes ao dia), alopurinol (100 mg ao dia), apixabana (5 mg, 2 vezes ao dia). Não houve novos episódios de descompensação clínica nos três meses que se passaram desde a alta hospitalar.

## Discussão

De acordo com a Diretriz Brasileira de IC Crônica e Aguda, ^3^ a sistematização de cuidados para a alta hospitalar em pacientes com IC descompensada inclui a resolução dos fatores precipitantes. Infecções, principalmente as pneumonias bacterianas, representam importantes causas de descompensação da IC. ^1^ Neste sentido, tem-se recomendado a vacinação contra pneumococo e vírus influenzae em pacientes com IC. Esta recomendação segue uma orientação dos EUA e países europeus, com clima mais temperado, onde a infecções graves por vírus influenzae são comuns. ^3^ Apesar destas infecções serem comuns no Brasil também, devemos ressaltar a proporção epidêmica que as arboviroses alcançaram em diferentes estados brasileiros. ^7^

A febre chikungunya é uma arbovirose transmitida por um alfavírus (CHIKV), e tem como vetores os mosquitos do gênero Aedes, sendo o *Ae. aegypti* o principal. ^10^ Foi documentada primeiramente na Tanzânia em 1952, e no Brasil o primeiro caso de transmissão autóctone foi registrado em 2014. ^10^ O nome chikungunya significa “andar curvado”, fazendo referência à marcante artralgia da doença, intensa e por vezes incapacitante, que pode durar de meses a anos. ^10^

Apesar da recente epidemia de febre chikungunya no Brasil e da grande prevalência de IC, não encontramos publicações citando esta virose como causa da agudização de IC crônica. Uma meta-análise recém-publicada ^6^ sugere que o sistema cardiovascular seja acometido em 54,2% dos casos de febre chikungunya, entretanto, devemos enfatizar que esta estatística baseou-se em relatos onde não houve padronização da definição deste acometimento, incluindo hipotensão, choque, arritmias, aumento de troponina e até miocardite aguda. ^6 - 8^ Com base nestes achados, os autores sugerem tropismo miocárdico pelo CHIKV, que, assim como o vírus da dengue, o parvovírus, o herpes vírus e o enterovírus, pode causar dano direto às células miocárdicas. ^6^

Paralelamente, as alterações hemodinâmicas características das infecções sistêmicas (como vasodilatação e taquicardia) podem ser suficientes para a descompensação clínica de pacientes com IC, gerando hipotensão e extravasamento de fluidos para o espaço extra-vascular. De fato, quando estes pacientes são infectados pelo CHIKV, pode haver descompensação clínica mesmo na ausência de miocardite.

Os sintomas da febre chikungunya geralmente surgem após um período de incubação de 1 a 12 dias. ^11^ A positividade para anticorpos IgM e IgG revela infecção recente ou atual, uma vez que os anticorpos IgM podem permanecer positivos por até 3 meses após a picada. O paciente descrito teve evolução clínica atípica, uma vez que a descompensação hemodinâmica ocorreu antes da artralgia característica da febre. Ainda assim, a ausência de outros fatores precipitantes, as sorologias positivas e a evolução temporal do quadro ( Figura 1 ) corroboram a hipótese de descompensação clínica por febre chikungunya no presente caso. Infelizmente, não foi possível realizar ressonância nuclear magnética cardíaca, pois a mesma não está disponível em nosso hospital. Ressaltamos que este exame, apesar de útil no diagnóstico de miocardite, não seria capaz de confirmar a hipótese de descompensação clínica por febre chikungunya.

Além do diagnóstico difícil, o presente caso caracterizou-se pelo desafio no manejo clínico. Como em outras arboviroses, o tratamento de pacientes com febre chikungunya tem sua base no controle álgico adequado, normalmente obtido com uso de anti-inflamatórios não esteroidais (AINEs) que não atuem como anti-agregantes plaquetários (como o ácido acetil salicílico). Entretanto, a disfunção miocárdica contra-indicou o uso de a AINEs ^3^ e por isso tivemos que optar por analgesia com opioides como o tramadol. Além disso, a plaquetopenia e o sangramento ativo (hematúria) impediram a continuidade da anticoagulação profilática no paciente, a despeito da indicação por fibrilação atrial crônica, aumentando o risco de evento tromboembólicos secundários à arritmia.

## Conclusão

Infecções virais, principalmente aquelas mais prevalentes em nosso meio, como a febre chikungunya, devem ser consideradas como fator de descompensação da IC em pacientes previamente estáveis sem outros fatores precipitantes claramente identificados.
