# Supplementary material for: Descompensação de Insuficiência Cardíaca por Arbovirose
Source: Arq Bras Cardiol. 2020 May 11;114(4 Suppl 1):19–22. [Article in Portuguese] doi: 10.36660/abc.20180316 (PMC8149109; doi:10.36660/abc.20180316)
Supplement: Translation

# Translation: Acute Decompensated Heart Failure due to Chikungunya Fever

**Authors:** Carolina Cunto de Athayde, Fabio Akio Nishijuka, Márcia Cavalcanti de Campos Queiroz, Monica Medeiros Luna, Jaime Lobo Figueiredo, Nadia Matias de Albuquerque, Sebastião Carlos Ribeiro de Castilho, Renata R. T. Castro

**Affiliations:** 1 Brazil Hospital Naval Marcilio Dias – Cardiologia, Rio de Janeiro, RJ - Brazil; 2 USA Brigham and Womens Hospital – Medicine, Boston – USA

**Keywords:** Heart Failure/physiopathology, Treatment Refusai, Pneumococcal Vaccine, Arbovirus Infections, Zika Virus Infection, ChiKungunia fever

## Introduction

Heart failure (HF) is a chronic condition with worldwide high and growing prevalence.^1 - 3^

It is extremely important to identify the cause of decompensated HF in order to manage cases correctly, given that identification makes it possible to implement specific treatment and prevent new hospitalizations. In Brazil, the main causes are poor adherence to medical treatment (30%) and infections (23%),^1^ mainly pulmonary bacterial ones.^4^ For this reason, patients with HF should receive the pneumococcal vaccine. Although it is less common, decompensation may occur due to viral infections, which justifies vaccination against influenza in these patients.^3^

Over the past years, diverse Brazilian cities have been affected by epidemics of arboviruses that had previously been considered rare, such as those caused by the Zika and the chikungunya viruses.^5^ These epidemics have drawn the scientific community’s attention, not only due to the number of patients affected, but mainly due to the common sequelae, such as microcephaly in children of pregnant women affected by the Zika virus and the disabling, chronic arthralgia secondary to chikungunya fever. Although there are case reports of myocarditis caused by arboviruses,^6 - 8^ little is known regarding the risks of compliactions when patients previously diagnosed with heart failure are affected. The high prevalence of HF worldwide and the high incidence of arboviruses in Brazil justify the following case report.

## Case Report

A 71-year-old retired male patient sought emergency service due to dyspnea during light exertion, which had progressively worsened over the past two days, evolving to resting dyspnea and paroxysmal nocturnal dyspnea after an episode of unmeasured fever the previous evening. He denied coughing, chest pain, dizziness, and syncope. The patient has previously been diagnosed with hypertensive/alcoholic cardiomyopathy, chronic non-dialytic renal failure, permanent atrial fibrillation, hyperuricemia, chronic obstructive pulmonary disease, and cholelithiasis. He suffered from alcoholism, and he was a former smoker (47 packs/year, having ceased six years before). He regularly took carvedilol (12.5 mg in the morning and 25 mg at night), hydralazine (25 mg, 3 times daily), amlodipine (5 mg daily), furosemide (40 mg, 4 times daily), digoxin (0.125 mg daily), apixaban (2.5 mg, 2 times daily), bamifylline (300 mg daily), and formoterol/budesonide (12 mcg + 400 mcg, 2 times daily). Upon admission, the patient presented blood pressure of 110/84 mmHg, heart rate of 86 bpm, respiratory rate of 26 rpm, and jugular venous distention at 30°. Pulmonary auscultation revealed universally audible vesicular murmurs, without adventitious sounds, and cardiac auscultation revealed an irregular rhythm, with normal heart sounds and no accessory sounds. The patient had lower limb edema (2+/4+), and there was no ascites on physical examination.

There was no clinical evidence of angina, new arrhythmias, or infection ( Table 1 ). The patient and his wife denied poor adherence to medical treatment, consumption of alcohol, or excessive salt or liquid intake. It was, therefore, not possible to identify precipitating factors for the clinical picture of decompensated HF. The patient’s admission electrocardiogram showed atrial fibrillation rhythm and left bundle branch block. There were no electrocardiographic alterations suggestive of myocardial ischemia. Chest radiography showed an increase in the cardiothoracic index, with slight pulmonary congestion and no pleural effusion or pulmonary consolidation. For the purpose of screening for infection, a urinary sediment test was performed, and the results were normal.

**Table 1:** – Laboratory results during hospitalization

Laboratory exams	Day of hospitalization				

	1	3	5	12	14
C-reactive protein (mg/dL)	3.00	2.90	2.56	10.60	10.30
Urea (mg/dL)	96	148	102	72	107
Potassium (mEq/L)	6.3	5.8	3.5	4.6	4.6
Creatinine (mg/dL)	2.0	3.6	2.2	1.6	1.9
Hemoglobin (g%)	14.9	16.3	14.0	14.1	13.0
Leukocytes (mil/mm^3^)	6.9	7.9	5.7	8.7	5.7
Platelets (mil/mm^3^)	106	135	87	80	111

The patient was admitted, classified as hemodynamic profile B^9^ , and he underwent treatment with intravenous diuretics ( Figure 1 ).

**Figure 1:** - Case report timeline

On the third day of hospitalization, the patient progressed with worsened renal function, with creatinine clearance (Cockroft-Gault) of 19ml/min (creatinine 3.0 mg/dL) and hyperkalemia (6.1 mEq/l). On account of this complication, digoxin was suspended. After five days, the patient reached hemodynamic profile A, and the physician opted to change the furosemide route of administration from intravenous to oral. The same day, the patient presented macroscopic hematuria, and anticoagulation was suspended. On the seventh day of hospitalization, the patient complained of mild arthralgia in his knees and elbows, which he associated with his position in bed. Notwithstanding use of dipyrone, the following day, the symptoms worsened to bilateral arthralgia, which was highly intense in the knees, ankles, wrists, and elbows, thus restricting the patient’s movement in bed. The patient did not present dyspnea or precordial pain, and he maintained hemodynamic profile A. Slight pain control was achieved with the regular use of tramadol, and, the following day, laboratory exams revealed thrombocytopenia (drop from 135,000 to 87,000 per mm^3^in 5 days, Table 1 ). The arthralgia and thrombocytopenia led to clinical suspicion of arbovirus, and serology was thus requested for dengue and chikungunya. The patient progressed with worsened peripheral and central congestion, with lower limb edema (3+/4+), jugular venous distention, hepatojugular reflux, bibasilar crackles, and square-wave systolic blood pressure response to the Valsalva maneuver. There were no signs of hypoperfusion, and the patient was again classified as hemodynamic profile B, and intravenous diuretic therapy was reinitiated.

On the fourteenth day of hospitalization, the thrombocytopenia, arthralgia, and pattern of congestion (returning to hemodynamic profile A) improved. Laboratory exams did not reveal electrolytic disorders ( Table 1 ) or alterations in liver function. The blood tests for chikungunya fever were positive (IgG and IgM). Thus, full anticoagulation with apixaban was reinitiated, and regular analgesia was changed to only if necessary. Throughout the entire hospitalization, the electrocardiographic pattern was maintained. The following day, the patient remained stable, maintaining a dry-warm hemodynamic profile, and he was discharged from the hospital with the following prescription: furosemide (40 mg, twice daily, carvedilol (25 mg, twice daily), atorvastatin (20 mg daily), formoterol + budesonide (12/400 mcg, twice daily), allopurinol (100 mg daily), apixaban (5 mg, twice daily). There were no new episodes of clinical decompensation during the three months following hospital discharge.

## Discussion

According to the Brazilian Guidelines on Chronic and Acute HF,^3^ systematizing care for discharging patients with decompensated HF from the hospital includes resolving precipitating factors. Infections, mainly pulmonary bacterial ones, represent important causes of decompensated HF.^1^ Accordingly, vaccination against pneumococcus and influenza viruses has been recommended for patients with HF. This recommendation is aligned with United State´s and European guidelines, which have more temperate climates, where serious influenza virus infections are common.^3^ Although these infections are also common in Brazil, it is necessary to emphasize the epidemic proportion that arboviruses have reached in diverse Brazilian states.^7^

Chikungunya fever is an arboviruses transmited by an alphavirus (CHIKV). Its vectors are mosquitoes of the *Aedes* genus, *Aedes aegypti* being the main one.^10^ It was first documented in Tanzania in 1952, and the first case of autochthonous transmission in Brazil was reported in 2014.^10^ The name chikungunya means “crooked walk,” referring to the pronounced arthralgia caused by the disease, which is intense and at times disabling and can last for months or years.^10^

Notwithstanding the recent chikungunya fever epidemic in Brazil and the high prevalence of HF, we have not found any publications citing this virus as the cause of chronic HF becoming acute. A recently published meta-analysis^6^ suggests that the cardiovascular system is involved in 54.2% of cases of chikungunya fever; it is, however, necessary to emphasize that this statistic is based on reports without any standardization of the definition of this involvement, including hypotension, shock, arrhythmias, increased troponin, and even acute myocarditis.^6 - 8^ Based on these findings, the authors suggest myocardial tropism due to CHIKV, which, like the dengue virus, parvovirus, herpes virus, and enterovirus, can cause direct damage to myocardial cells.^6^

Similarly, the hemodynamic changes that are characteristic of systemic infections (such as vasodilation and tachycardia) may be sufficient for clinical decompensation to occur in patients with HF, generating hypotension and fluid leakage into extra-vascular space. In fact, when these patients are infected by CHIKV, clinical decompensation may occur, even in the absence of myocarditis.

Symptoms of chikungunya generally appear after an incubation period of one to twelve days.^11^ Positivity for IgM and IgG antibodies indicates recent or current infection, given that IgM antibodies may remain positive for up to three months after the bite. The patient described had an atypical clinical progression, considering that hemodynamic decompensation occurred before the arthralgia characteristic of the fever. Nonetheless, the absence of other precipitating factors, the positive blood tests, and the clinical picture’s evolution over time ( Figure 1 ) corroborate the hypothesis of clinical decompensation due to chikungunya fever in the present case. Unfortunately, it was not possible to perform cardiac nuclear magnetic resonance, because it was not available at our hospital. It is worth emphasizing that the exam, although useful for diagnosis myocarditis, would not have been able to confirm the hypothesis of clinical decompensation due to chikungunya fever.

In addition to being difficult to diagnose, the present case was characterized by challenges in clinical management. As with other arboviruses, treatment of patients with chikungunya fever is based on adequate pain control, which is normally achieved through the use of nonsteroidal anti-inflammatory drugs (NSAID) that do not have an antiplatelet aggregation effect (such as acetylsalicylic acid). However, myocardial dysfunction counterindicated the used of NSAIDF, and, for this reason, it was necessary to opt for analgesia with opioids like tramadol. Furthermore, thrombocytopenia and active bleeding (hematuria) impeded continuation of prophylactic anticoagulation in the patient, in spite of the indication for chronic atrial fibrillation, thus increasing the risk of thromboembolic events secondary to arrhythmia.

## Conclusion

Viral infections, especially those that are most prevalent in Brazil, such as chikungunya fever, should be considered as factors of decompensated HF in patients who were previously stable without any other clearly identified precipitating factors.
